# Vapoenucleation of the prostate using a high-power thulium laser: a one-year follow-up study

**DOI:** 10.1186/s12894-015-0032-7

**Published:** 2015-05-09

**Authors:** Ching-Hsin Chang, Tzu-Ping Lin, Yen-Hwa Chang, William JS Huang, Alex TL Lin, Kuang-Kuo Chen

**Affiliations:** Department of Urology, Taipei Veterans General Hospital, Taipei, Taiwan; Department of Urology, National Yang-Ming University School of Medicine, Taipei, Taiwan; Shu-Tien Urological Science Research Center, Taipei, Taiwan; Department of Urology, Taipei Medical University Hospital, Taipei, Taiwan; Graduate Institute of Medical Sciences, College of Medicine, Taipei Medical University, Taipei, Taiwan

**Keywords:** Benign prostate enlargement, Quanta thulium surgical laser system, Thulium laser, Vapoenucleation

## Abstract

**Background:**

Prostate vaporization and enucleation is a novel treatment option for bladder outlet obstruction caused by benign prostate enlargement. This surgical technique, however, has not yet been standardized. We present our findings of using a high-power thulium laser to accomplish vapoenucleation of the prostate (ThuVEP).

**Methods:**

We prospectively collected and analyzed data from 29 patients who underwent ThuVEP between August 2010 and May 2012. The control group included 30 patients who underwent traditional transurethral resection of the prostate (TURP). Operative variables, patient profiles, preoperative and postoperative urine flow rates, prostate volume (measured using transrectal ultrasonography), and the international prostate symptom score (IPSS) were recorded and analyzed using a two-tailed Student’s *t*-test and analysis of variance.

**Results:**

The ages (mean ± SD) of the patients were 76.1 ± 9.4 and 72.6 ± 7.4 years (*p* = 0.28) in the ThuVEP and TURP groups, respectively. The average urinary flow rates before and 12 months after the operation (volume/maximum flow/average flow) were 243.3/10.5/5.0 and 302.8/17.6/9.4 (in mL, mL/s, mL/s, respectively) in the ThuVEP group and 247.2/10.8/4.6 and 369.9/20.8/12.0, respectively, in the TURP group. Preoperative and postoperative IPSSs were 17.1 ± 5.0 and 6.5 ± 3.8, respectively, in the ThuVEP group and 18.2 ± 4.5 and 6.2 ± 3.3, respectively, in the TURP group. The mean ratio of the estimated postoperative residual prostate volume to the preoperative total volume was 0.47 (*p* = 0.449) in both groups. The overall complication rate was 20.7% in the ThuVEP group and 30.0% in the TURP group.

**Conclusions:**

One year of follow-up showed that ThuVEP and TURP effectively alleviated subjective and objective voiding symptoms with a low rate of complications. Thus, vapoenucleation using a high-power laser is feasible in elderly patients.

**Trial registration:**

ISRCTN registry with study ID ISRCTN52339705. Date assigned: 06/03/2015.

## Background

Benign prostate enlargement (BPE) with lower urinary tract symptoms is a commonly observed condition in the daily clinical practice of urologists, especially those treating an aging male population. Surgical intervention is indicated for patients who develop complications associated with bladder outlet obstruction. Acute urinary retention is the most frequently reported complication, occurring in 0.5–6.5% of patients [1]. The likelihood of a male patient requiring transurethral resection of the prostate (TURP) increases by 6, 14, and 18 times with each decade of life after 59 years of age [2].

Surgical interventions and second-line measures are the treatments of choice for high-risk patients [3]. Surgical intervention is believed to have the most significant influence on the natural course of BPE and on preventing complications [4]. Surgical interventions include TURP (monopolar or bipolar) and laser treatment of the prostate, which includes holmium laser enucleation of the prostate (HoLEP), green light laser, and thulium laser [5].

Several studies have shown that TURP is the most common, widely performed, effective, and cost-efficient treatment to date [5-7]. Although TURP is associated with low morbidity [8], we should explore techniques other than TURP that have even lower morbidity rates.

HoLEP is the most commonly performed surgical intervention in recent decades, and the improvements in urodynamic parameters with this technique are comparable to those obtained with other techniques when performed by experienced surgeons [9]. Krambeck et al. showed a satisfactory outcome and low morbidity with HoLEP [10].

The over-deobstruction achieved by HoLEP, however, might induce postoperative incontinence at an early stage [10]. Because of a steep learning curve, the adoption rate for this technique is low, and only a few studies on large series of patients have been performed in limited centers [11,12]. The learning curve may be prolonged, and 50 cases are required for a surgeon to obtain an outcome that is comparable to that reported in the literature [13].

Thulium laser has several theoretical advantages over the holmium laser (e.g., rapid vaporization and coagulation ability, improved spatial beam quality, precise tissue incision) [14,15]. Bach et al. presented the first study on thulium:yttrium aluminum garnet (Tm:YAG) laser prostatectomy using the vaporesection technique with a 70-W Tm:YAG laser [16]. Shao et al. reported less blood loss in the Tm:YAG group, and the outcome in this group was similar to that observed in the group that underwent enucleation of the prostate using the Ho:YAG laser [17].

Xia et al. reported the first prospective randomized study comparing thulium laser resection with the prostate-tangerine technique (TmLRP-TT) and the standard TURP for symptomatic BPE with a 50-W instrument [18]. Both groups showed significant improvement in subjective symptom scores and urodynamic parameters. TmLRP-TT was significantly superior to TURP in terms of duration of catheterization, duration of hospitalization, and decrease in hemoglobin level [19]. The aforementioned thulium laser series with the Tm:YAG laser (RevoLix®; LISA Laser Products, Katlenburg, Germany) showed promising results. With the advancements in their technology, thulium lasers with higher energy output are now available. An example is the Quanta System Cyber Th:YAG laser. This new generation of Th:YAG lasers offers a maximum energy output of 150 W.

To our knowledge, the present study is the first prospective nonrandomized trial of vapoenucleation using the Cyber Th:YAG laser versus TURP with a 1-year follow-up.

## Methods

After receiving approval from the institutional review board of the Taipei Veterans General Hospital (VGHIRB No. 201007014IC), we recruited patients between August 2010 and May 2012 who had BPE that required surgical intervention. All consents were obtained with the method of written. The study was designed as a prospectively nonblind randomized trial. It included 59 patients. The Th:YAG laser vapoenucleation (ThuVEP) group included 29 patients and the TURP group had 30 patients. Informed consents for participation were obtained from the participants.

The inclusion criteria were an international prostate symptom score (IPSS) >7, maximum urinary flow rate (Qmax) <15 mL/s, and normal level of age-specific prostate-specific antigen (PSA) [20]. Patients with abnormal levels of age-specific PSA or positive findings on digital rectal examination underwent transrectal ultrasonography (TRUS)-guided biopsy to rule out prostate cancer. Ten patients underwent TRUS-guided biopsy before the operation. To ensure intraoperative safety, we asked the patients to discontinue all anticoagulants except low-dose aspirin.

Before the operation, we evaluated the IPSS and quality of life score (QoLs) in each patient. Each was administered the five-item international index of erectile function (IIEF-5) questionnaire. Prostate volume (V1) was measured using TRUS and the prolate ellipsoid volume formula. The postvoid residual (PVR) urine volume was measured using bladder scans and uroflowmetry (UFR).

The patients were placed in the lithotomy position under spinal anesthesia. A single surgeon (T.P.L.) performed all of the ThuVEP procedures. TURP was performed by three surgeons. We used 150-W thulium lasers (Quanta Thulium Surgical Laser System: Cyber TM; Quanta Systems, Solbiate Olona, Italy) coupled with a 26-Fr resectoscope sheath (No. A22040A; Olympus, Tokyo, Japan) for this procedure. Energy was delivered via 550-mm end-firing fibers.

**Figure 1:**
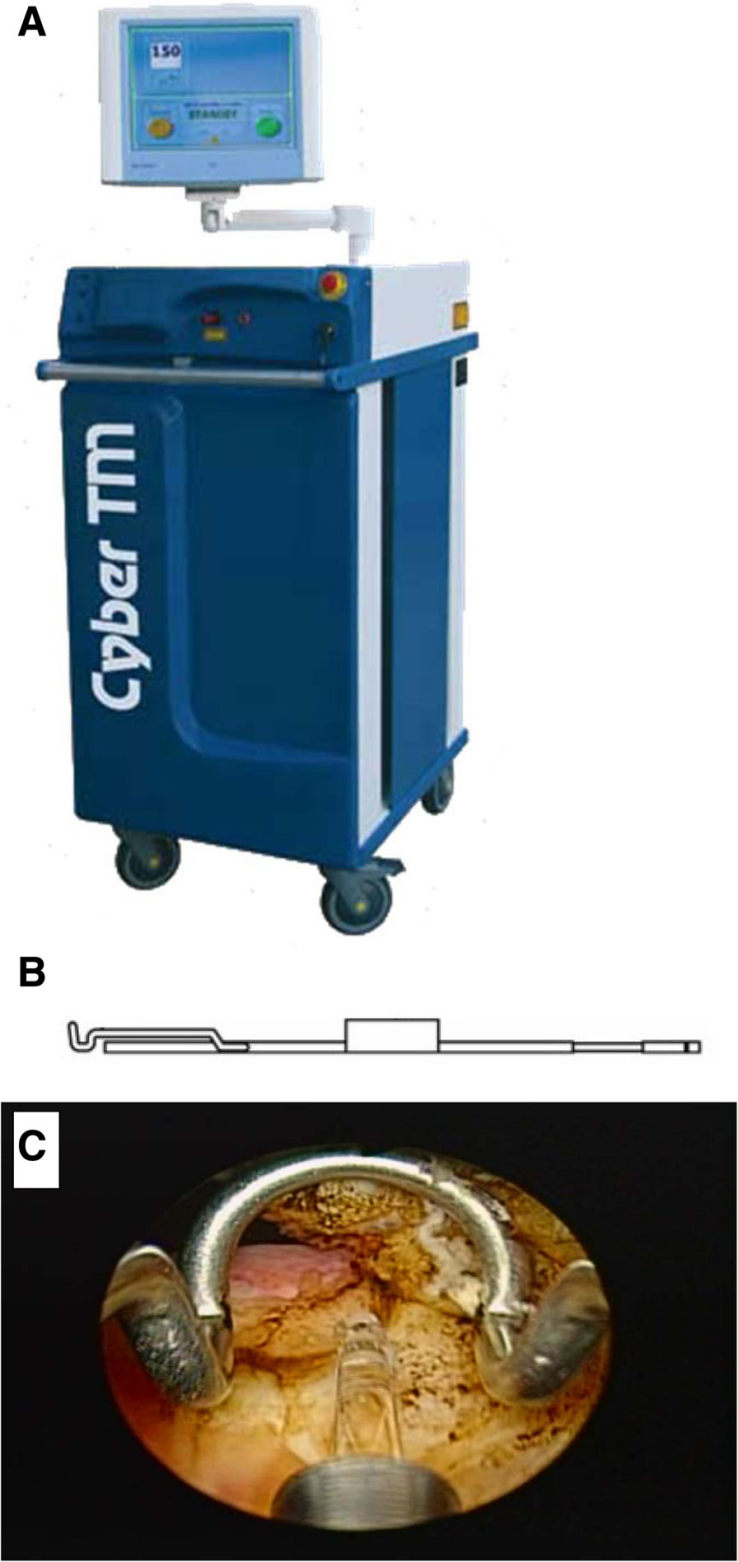
Overall laser system used. **A**: Quanta thulium surgical laser system. **B**: View from the guiding tube. **C**: Procedure for laser enucleation with the guiding tube. The median lobe was elevated using the guiding tube. Thus, the surgical plan used tension to further facilitate precise incision of the surgical capsule.

The technique utilized in the ThuVEP group was similar to that used for trilobular HoLEP [21]. Briefly, we initiated the procedure by making two Turner–Warwick-like incisions in the 5 and 7 o’clock direction from the bladder neck to the level of the verumontanum. The incision continued to the surgical capsule. Then, we made a third incision from the bladder neck to the level of the verumontanum (the incision was not too distal) in the 12 o’clock direction. The three lobes were then enucleated starting at the median lobe followed by the right and left lobes. We did not perform a high degree of blunt enucleation by using the beak of the resectoscope. Instead, we used laser energy to incise and connect the incisions performed previously. This procedure was facilitated using a guiding tube (No. A00561A; Olympus) made specifically to elevate the incised prostate from the plane between the adenoma and the surgical capsule of the prostate (Figure 1). After all three lobes were enucleated from the prostate capsule, the three adenomas were pushed into the bladder and morcellated with a mechanical tissue morcellator, then the tissue is evacuated from bladder. At the end of this procedure, the operation site was irrigated with saline in all patients.

In the TURP group, TURP was performed using a standard tungsten wire loop with a cutting current of 160 W and a coagulating current of 80 W. All of the prostate chips were removed using a Toomey evacuator.

At the end of both procedures, we inserted a 22-Fr triple-lumen catheter into the bladder and initiated irrigation until hematuria had decreased to a sufficient degree. We performed postoperative histological examination of all the tissues retrieved. Typically, the urethral catheter was removed early on postoperative day 2 or 3 if severe bleeding was not observed in the urine. The voiding pattern was monitored for 1 day, and the patient was discharged home.

The perioperative primary outcomes measured in the two groups included the operative time (interval when the resectoscope sheath was within the urethra), weight of the resected tissue (actual weight of the tissue retrieved), decreased hemoglobin level, decreased serum sodium level, duration of postoperative catheterization, and postoperative hospital stay. We evaluated the IPSS, QoLs, Qmax, and PVR urine in both groups at discharge and at 3, 6, and 12 months after the operation. We recorded all perioperative complications. The IPSS was obtained from the questionnaire and included two subscores: voiding symptoms (incomplete emptying, intermittence, weak stream, straining to void) and storage symptoms (frequency, urgency, nocturia). In addition, preoperative and postoperative sexual function was evaluated from the IIEF-5 [22].

At 6 months after the operation, the prostate volume of each patient was measured using a TRUS procedure using an ultrasonography machine (BK Medical, Herlev, Denmark) with a 7.5-MHz TRUS probe. The estimated residual prostate volume (V2) was calculated as the volume of the entire gland using a prolate elliptical formula (height × width × length × π/6) minus the central defect (also calculated using the prolate elliptical formula). The estimated residual prostate volume ratio was calculated as V2/V1.

All measurement data for the two groups were statistically analyzed using a two-tailed Student’s *t* test. The data are presented as the mean ± standard deviation (SD). The scoring and questionnaire results were analyzed using analysis of variance (ANOVA). Statistical significance was defined as *p* < 0.05.

## Results

### Baseline characteristics

Most of the baseline characteristics in the two groups showed no differences (Table 1). However, the PVR in the ThuVEP group was higher than that in the TURP group (138.6 ± 127.7 vs. 90.9 ± 66.5, *p* = 0.040). The mean age of the patients in our study was higher than that reported in previous studies [19]. TRUS-guided biopsy was performed in 15.3% of the patients before the procedure because of increased levels of age-specific PSA.

**Table 1:** **Baseline characteristics for the groups**

**Characteristic**	**ThuVEP**	**TURP**	***p***
Number	29	30	
Age (years)	76.1 ± 9.4	72.6 ± 7.4	0.280
Anticoagulant*	15(51.7%)	6(20.0%)	0.011
BMI	23.9 ± 2.7	23.7 ± 3.4	0.281
PSA (ng/mL)	5.0 ± 5.4	8.3 ± 7.9	0.076
TRUS estimated weight (g)	57.2 ± 25.1	64.7 ± 32.5	0.758
IPSS	17.1 ± 5.0	17.8 ± 4.3	0.674
Charlson co-morbidity index	1.0 ± 1.0	1.6 ± 2.7	0.639
Qmax (mL/s)	10.5 ± 4.9	10.8 ± 4.7	0.728
PVR vol (mL)*	138.6 ± 127.7	90.9 ± 66.5	0.040

*Statistical difference.

BMI, body mass index; PSA, prostate-specific antigen; TRUS, transrectal ultrasound; IPSS, international prostate symptom score; Qmax, maximum urinary flow rate; PVR, post-void residual volume; ThuVEP, vapoenucleation of the prostate using a high-power thulium laser; TURP, transurethral resection of the prostate.

### Perioperative data

The resected tissue was heavier in the TURP group than in the ThuVEP group (37.4 ± 23.0 vs. 21.3 ± 14.3 g, *p* = 0.024), although the estimated residual prostate volume ratio (0.47 ± 0.17 vs. 0.47 ± 0.20, *p* = 0.449) was the same in the two groups. The duration of catheterization (1.8 ± 0.5 vs. 2.3 ± 0.5 days, *p* = 0.001) and postoperative hospital stay (3.0 ± 0.9 vs. 3.4 ± 0.7 days, *p* = 0.032) were shorter for the ThuVEP group than for the TURP group. Other variables, such as total duration of hospitalization, decrease in hemoglobin levels (0.5 ± 1.3 vs. 0.5 ± 1.1, *p* = 0.844), and decrease in serum sodium levels (0.3 ± 2.4 vs. 1.6 ± 2.0, *p* = 0.468) were not statistically different (Table 2). In all, 96.3% patients in the ThuVEP group and 93.3% in the TURP group completed the 1-year follow-up study.

**Table 2:** **Perioperative data**

**Parameter**	**ThuVEP**	**TURP**	***p***
Resected weight (g)*	21.3 ± 14.3	37.4 ± 23.0	0.024
Preoperative hemoglobin level (g/dL)	12.9 ± 1.7	13.3 ± 1.6	0.587
Decrease in hemoglobin level (g/dL)	0.5 ± 1.3	0.5 ± 1.1	0.844
Decrease in serum sodium level (mmol/L)	0.3 ± 2.4	1.6 ± 2.0	0.468
Duration of catheterization (day)*	1.8 ± 0.5	2.3 ± 0.5	0.001
Total duration of hospitalization (days)	5.2 ± 1.9	5.2 ± 0.8	0.203
Postoperative duration of hospitalization (days)*	3.0 ± 0.9	3.4 ± 0.7	0.032
Estimated residual prostate volume ratio (V2/V1)	0.47 ± 0.17	0.47 ± 0.20	0.449
PSA ratio (before the operation/12 months after the operation)	2.4 ± 2.9	1.6 ± 1.0	0.180

ThuVEP, vapoenucleation of the prostate using a high-power thulium laser; TURP, transurethral resection of the prostate; PSA, prostate-specific antigen.

The duration of catheterization and postoperative hospitalization showed statistical differences. The estimated residual prostate volume (V2) was calculated as the volume of the entire gland using prolate elliptical formula (height × width × length × π/6) minus the central defect (also calculated using the prolate elliptical formula). The estimated residual prostate volume ratio was calculated as V2/V1.

(*Statistical difference).

### Follow-up of QoLs

**Figure 2:**
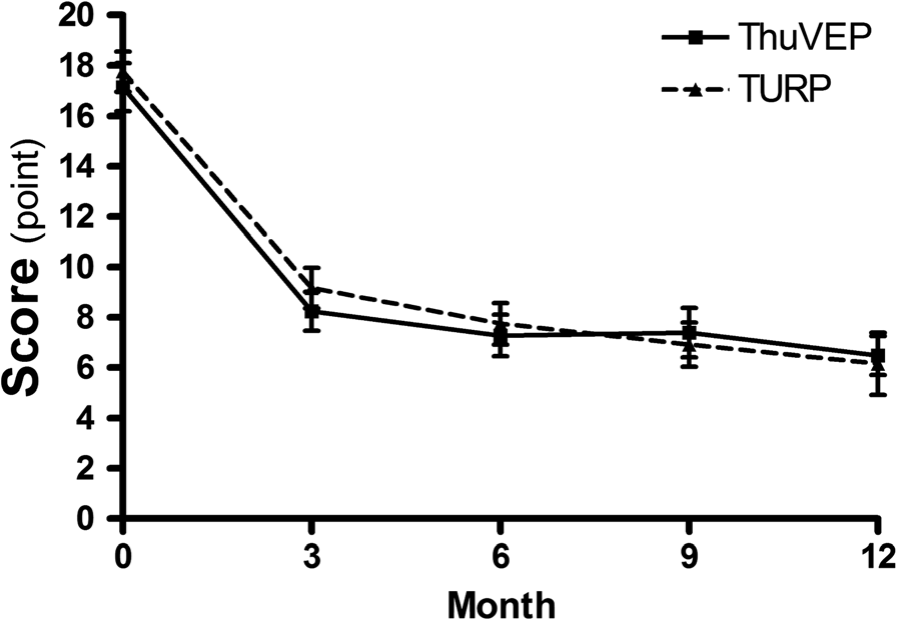
International prostate symptom score (IPSS). No statistical differences were observed between the two groups.

The IPSS and QoLs in both groups had decreased significantly postoperatively (Figure 2). The symptoms and QoLs, however, began to improve and continued up to 12 months after the operation. The IPSS, including total scores, subscores of voiding symptoms, and subscores of storage symptoms, all displayed a similar trend of continuing to improve. There were no statistically significant differences in these values between the two groups (*p* = 0.551).

### Follow-up of UFR and PVR urine volume

**Figure 3:**
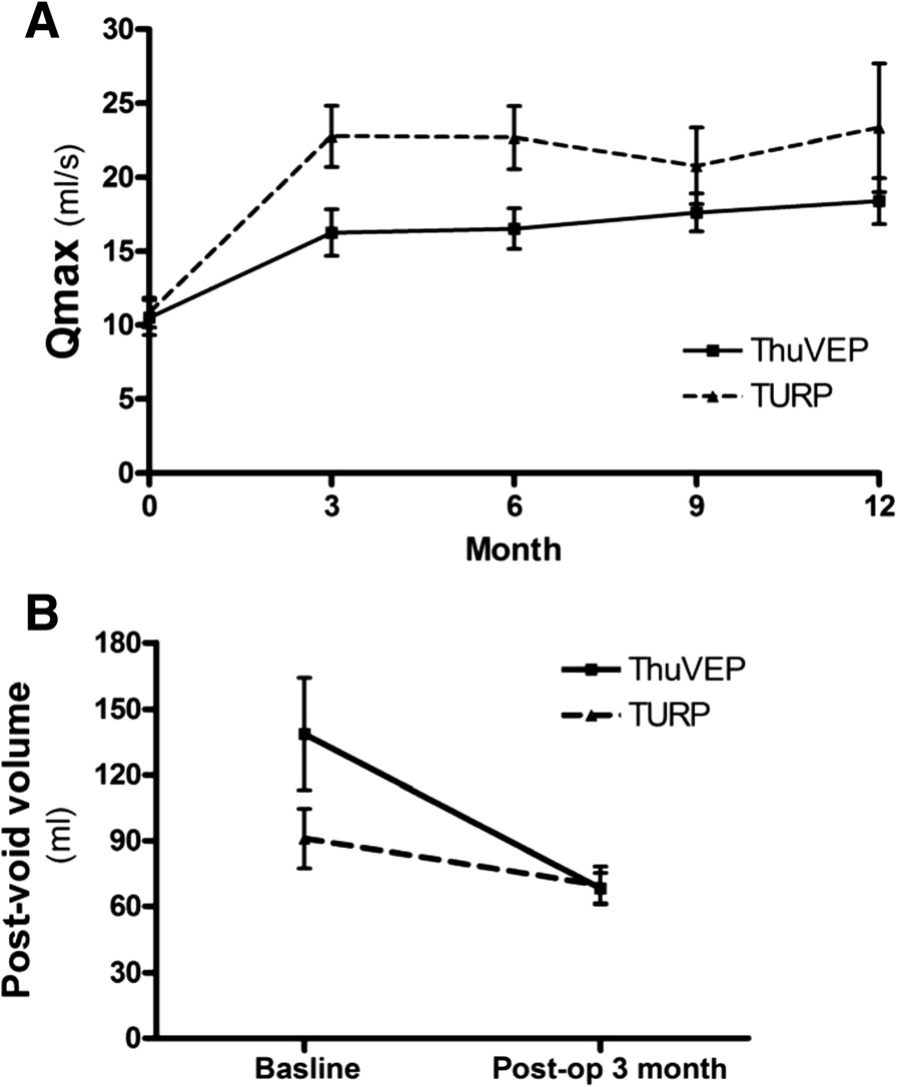
Uroflowmetry (UFR). **A**: Maximum urinary flow rate (Qmax) according to the UFR test data. No statistical differences were observed in the two groups. **B**: Postvoid residual volume in the UFR test data. All variables improved significantly in the two groups after the operation without statistical differences at later follow-up examinations.

All UFR variables – voided volume, Qmax, mean flow rate – improved significantly in both groups after the operation (not all of the data are shown, Figure 3). The preoperative PVR urine volume was significantly higher in the ThuVEP group. The postoperative PVR urine volume markedly decreased in both groups, with no difference observed in the PVR urine volumes 3 months after the operation in the two groups (68.2 ± 37.5 vs. 69.5 ± 47.9, *p* = 0.648).

### Complications

We used the modified Clavien classification system for reporting TURP-related complications in our patients, as proposed by Smith and Patel [23]. Six complications were recorded among the 29 patients in the ThuVEP group: 6.9% patients had grade I complications, and 13.8% had grade II complications. None of the patients exhibited grade III–V complications. Nine complications were recorded in the 30 TURP group patients. Grade I complications were observed in 1.3% of patients and grade II in 26.7%. No higher-grade complications were observed in this group (Table 3).

**Table 3:** **Modified Clavien classification system for reporting complications of TURP**

**Criteria**	**ThuVEP (N = 29)**	**TURP (N = 30)**
Grade 1		
Hematuria clot retention requiring bladder irrigation/clot evacuation/catheter traction	0	1 (3.3%)
Catheter block because of retained TUR chip	0	0
Failed trial without catheter with acute urinary retention requiring bedside recatheterization	2 (6.9%)	0
Transient increase in the serum creatinine level	0	0
Lower urinary tract infection requiring antibiotics	0	0
Grade 2		
Hemorrhage/hematuria requiring transfusion	4 (13.8%)	8 (26.7%)
Urinary tract infection with bacteremia requiring antibiotics	0	0
Supraventricular tachycardia requiring antiarrhythmia drugs	0	0
Pulmonary embolism requiring anticoagulants	0	0
Grade 3		
Extraperitoneal fluid collection caused by subtrigonal catheter requiring endoscopic catheter repositioning and surgical drainage	0	0
Grade 4		
Acute myocardial infarction requiring admission to the ICU	0	0
TUR syndrome requiring admission to the ICU	0	0
Grade 5		
Death	0	0
Total	6	9

ThuVEP, vapoenucleation of the prostate using a high-power thulium laser; TURP, transurethral resection of the prostate.

## Discussion

Surgical techniques used to treat bladder outlet obstruction include Tm:YAG vaporization of the prostate (ThuVAP), Tm:YAG vaporesection of the prostate (ThuVaRP), ThuVEP, and Tm:YAG laser enucleation of the prostate (ThuLEP) [24].

The wavelength of thulium laser matches that of the water absorption peak in tissue at 1.92 mm, which results in sufficient hemostasis, a clear visual field, and rapid incision of the tissues with little thermal damage. Fried et al. reported that continuous-wave 50-W thulium fiber laser vaporized prostate tissue at a rate of 0.45 g/min [8].

Because of the high vaporization inherent in laser–tissue interaction with thulium-YAG laser, the true resected prostate volume was difficult to determine compared to that with other enucleation prostatectomies or TURP. To address this issue, we prospectively measured the prostate volume by TRUS. The estimated residual prostate volume was obtained by subtracting the volume of the entire prostate from that of the central defect, which were calculated using the values of the outer and inner dimensions, respectively. Although the prostate volume differed significantly after resection, the residual volumes after both approaches (ThuVEP and TURP) were not significantly different. The equal ratio of residual prostate volume was supported by equal symptom improvement and urodynamic improvement. Thus, the ratio of the postoperative prostate volume to the preoperative prostate volume that was estimated from TRUS-guided biopsy samples may better predict symptom alleviation or urodynamic improvement than tissue resected when ThuVEP is utilized.

HoLEP involves a three-lobe technique (median lobe and lateral lobes) for enucleation of the prostate [21]. ThuLEP was introduced by Herrmann et al. [25]. Unlike other techniques that involve determination of the surgical capsule at the apex, this technique involves apical incision of the prostatic tissue down to the surgical capsule. This approach is further facilitated by the superior Th:YAG–tissue interaction and higher vaporizing property. This approach did not compromise the deobstruction as shown by improved urodynamic parameters and alleviated symptoms. Also, this trend toward improvement was still ongoing at the 12-month follow-up.

The perioperative morbidity associated with TURP decreased when the surgical equipment was modified. However, the possibility of TUR syndrome, a common complication, continues to exist. Our study showed that the complication rate, blood transfusion rate, and decrease in the serum sodium and hemoglobin levels were lower with ThuVEP than with other laser instruments or TURP [26].

Laser surgery has been recommended because there is less risk of sexual dysfunction. Xia et al. reported that 3.8% patients in the TmLRP-TT group and 14.6% patients in the TURP group had slightly reduced erectile function at 12 months postoperatively [19]. Preoperatively, about 50% patients who underwent TmLRP-TT and TURP had not had erections sufficient for intercourse [19]. Because of the advanced age of the patients in our study, we did not have a sufficient number of potent patients before the operation in either group to assess this point. Therefore, we were unable to compare the theoretical complications of TUR surgery.

In previous studies, the intraoperative and postoperative complications covered a wide extent of damage and severity: clot retention; significant hematuria that prolongs hospitalization; open cystotomy to remove adenomas; myocardial infarction and atrial fibrillation that require cardioversion; morcellator bladder injury; cerebral vascular accident; sepsis [10]. We used the modified Clavien classification system to report and systematically analyze the complications according to treatment [27]. This method has been adopted in various surgical disciplines as it has improved detection and avoided observation bias. Masumori et al. was the first to use the Clavien classification system to report complications of TURP. The overall perioperative morbidity was 15.8%, including grade I (59.1%) and II (29.5%) complications, those requiring interventions (2.3%, grade III), intensive care unit (ICU) admission (6.8%, grade IV), and one death (0.5%, grade V) [6].

We used the modified Clavien classification system to classify our patients (Table 3). There were no high-grade (grades III–V) complications in either group in our study.

Age and the Charlson co-morbidity index are independent and highly significant (*p* < 0.001) predictors for mortality [7]. Use of oral anticoagulants was regarded as an independent factor for the outcome of TURP as it was associated with significantly longer hospitalization and higher rates of bladder clots, blood transfusions, late hematuria, and thromboembolic events [28].

We prospectively recorded the above factors for our patients (Table 1). A total of 35.6% of the patients in our study took anticoagulants. These patients tended to be older and had larger prostates with more tissue resected. The blood transfusion rate in this study was higher than that in our previous study, increasing from 2.2% to 4.2% [19,26]. There was a minimal difference, however, in the hemoglobin level before and after the operation (around 0.5 g/dL). These findings indicate that although the thresholds for blood transfusion were low, they would be permissible in these older, fragile patients.

We conducted a nonrandomized controlled study to compare ThuVEP and TURP. We prospectively collected the cohort data and systemically evaluated the Charlson index for co-morbidity and complications reported according to the modified Clavien classification. Our results showed that ThuVEP obtained equal and durable symptomatic and urodynamic improvements at the 12-month follow-up. Preoperative and postoperative TRUS evaluation of prostate size indicated that a uniform amount of tissue was excised. This was especially important in the ThuVEP group because the vaporization rate of the tissue is high using thulium laser. Thus, the tissue resected using ThuVEP cannot represent the exact volume of the resected prostatic tissue.

Our study has several limitations. Most of the procedures were performed by a single surgeon who was responsible for the entire ThuVEP group. Three surgeons were responsible for the TURP group (T.P.L., Y.H.C., W.J.H.). Thus, a comparison of these two groups may be subjected to bias. However, because all participating surgeons had engaged in their clinical practices for more than 10 years, they had accrued surgical experience with 50 patients or more per year. Thus, the operator-related bias was limited. Also, the follow-up duration was not sufficiently long. The outcome of the procedure may differ after a longer follow-up. Finally, our study was not randomized, which lowers the level of evidence-based medicine. Thus, large-scale, prospective, and randomized studies are required to eliminate the possible bias inherent to a nonrandomized study design.

## Conclusions

The outcome of high-energy (150 W) ThuVEP in terms of symptom alleviation and improved urodynamic parameters was similar to that obtained using TURP. This laser procedure is well tolerated, and enables efficient excision and rapid organic vaporization. The results indicate the feasibility of high-energy Th:YAG laser vapoenucleation for the treatment of benign prostate hyperplasia.

## Abbreviations

ThuVEP: Vapoenucleation of the prostate using high-power thulium laser
TURP: Transurethral resection of the prostate
ANOVA: Analysis of variance
BPE: Benign prostate enlargement
Tm: Thulium
YAG: Yttrium aluminum garnet
PVR: Postvoid residual
UFR: Uroflowmetry
